# Interleukin-27 re-educates intratumoral myeloid cells and down-regulates stemness genes in non-small cell lung cancer

**DOI:** 10.18632/oncotarget.2797

**Published:** 2015-01-03

**Authors:** Irma Airoldi, Maria Grazia Tupone, Silvia Esposito, Marco V. Russo, Giulia Barbarito, Giuseppe Cipollone, Emma Di Carlo

**Affiliations:** ^1^ Laboratory of Oncology, Istituto “Giannina Gaslini”, Genova 16147, Italy; ^2^ Anatomic Pathology and Molecular Medicine, Department of Medicine and Sciences of Aging, “G. d'Annunzio” University, Chieti 66100, Italy; ^3^ Ce.S.I. Biotech, Aging Research Center, “G. d'Annunzio” University Foundation, Chieti 66100, Italy; ^4^ Department of Experimental and Clinical Sciences, “G. d'Annunzio” University, Chieti 66100, Italy; ^5^ General and Thoracic Surgery, “SS Annunziata” Hospital, Chieti 66100, Italy

**Keywords:** cytokines, lung cancer, tumor microenvironment, inflammation, immunotherapy

## Abstract

Current therapies for Non-Small Cell Lung Cancer (NSCLC) still fail to significantly increase its survival rate. Here we asked whether Interleukin(IL)-27, which has revealed powerful antitumor activity and is toxicity-free in humans, is a promising therapeutic choice for NSCLC patients.

IL-27's effects were tested on Adenocarcinoma (AC) and Squamous Cell Carcinoma (SCC) cell lines and xenograft models. IL-27Receptor(R) expression was assessed in lung tissues from 78 NSCLC patients.

*In vitro*, IL-27 was ineffective on cancer cell proliferation or apoptosis, but fostered CXCL3/GROγ/MIP2β expression. *In vitro* and *in vivo*, IL-27 down-regulated stemness-related genes, namely *SONIC HEDGEHOG* in AC cells, and *OCT4A*, *SOX2*, *NOTCH1*, *KLF4* along with *Nestin*, *SNAI1/SNAIL*, *SNAI2/SLUG* and *ZEB1*, in SCC cells. *In vivo*, IL-27 hampered both AC and SCC tumor growth in association with a prominent granulocyte- and macrophage-driven colliquative necrosis, CXCL3 production, and a reduced pluripotency- and EMT-related gene expression. Myeloablation of tumor-bearing hosts mostly abolished IL-27's antitumor effects. In clinical samples, IL-27R expression was found in AC, SCC, pre-cancerous lesions and tumor infiltrating myeloid cells, and correlated with advanced stages of disease.

Our data suggest that even immunocompromised or advancer NSCLC patients may benefit from IL-27's antitumor properties based on its ability to drive myeloid cells towards antitumor activities, and down-regulate stemness- and EMT-related genes in cancer cells.

## INTRODUCTION

Lung cancer is the leading cause of cancer induced mortality worldwide [1, 2]. Non-small cell lung cancers (NSCLC), particularly, adenocarcinoma (AC) and squamous cell carcinoma (SCC), are the most frequently diagnosed histotypes [1, 3]. New targeted therapies have been developed and some are currently used to treat advanced AC, but are unsuitable for SCC [4, 5]. Moreover, not all AC patients benefit from these treatments [5]. Development of effective and well tolerable immunotherapies to replace or be combined with surgery, radiotherapy or personalized treatments may be of great value. In the last few years, Interleukin(IL)-27, a member of the IL-12 family of cytokines, with important roles in both innate and adaptive immunity [6, 7], has revealed potent antitumor effects in the form of anti-proliferation, anti-angiogenesis, and immune system stimulation in a variety of tumors [8–10]. Its over-expression in murine Lewis lung carcinoma line 1 (LLC1) cells induces a specific cytotoxic T cell and antibody response *in vivo*, and also activates non-immunological mechanisms reducing cancer cell motility and migration [11]. Inhibition of AC cell migration, together with down-regulation of pro-angiogenesis genes by IL-27 has also been reported in the human A549 AC cell line [12]. Moreover, murine IL-27 gene-transfected LLC1 cells have been used to generate an autologous cell vaccine boosting an efficient T lymphocyte activation and IFNγ production [13]. However, definitive *in vivo* proof of IL-27's efficacy in pre-clinical models of human lung AC and SCC is still lacking.

We here investigate the *in vitro* and *in vivo* effects of IL-27 on the regulation of angiogenesis-, stemness- and epitelial-mesenchymal transition (EMT)-related genes in human AC and SCC cell lines and lung tumors grown in B/T cell deficient mice. Furthermore, by means of molecular biology and immunohistochemical studies, we have assessed IL-27Receptor(R) expression in lung cancer samples and analyzed the rationale for a future IL-27 application in the clinical setting of NSCLC.

## RESULTS

### Human lung AC and SCC cell lines express IL-27R and respond to IL-27 up-regulating CXCL3 expression and down-modulating stemness- and EMT-related genes

Since AC and SCC are the most common histotypes of lung cancers, ~85% of NSCLC [1, 3], we looked to see whether IL-27 acts as an antitumor agent in these forms.

**Figure 1:**
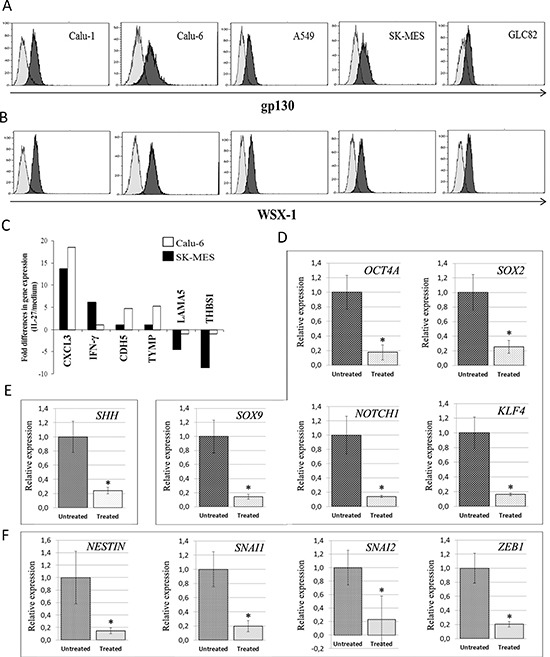
Expression of IL-27R in Human Lung Carcinoma Cell Lines, and IL-27's Effects on Angiogenesis, Stemness- and EMT-Related Gene Expression in Calu-6 and SK-MES Cell Lines Expression of gp130 **(A)** and WSX-1 **(B)** was analyzed in human lung carcinoma cell lines by flow cytometry. Open profile: gp130 (top) and WSX-1 (bottom) staining. Dark profile: isotype matched mAb staining. The mean percentage of gp130 expression was 65% in SK-MES and 64% in Calu-1 cells, 68% in Calu-6, 44% in GLC82 and 26% in A549 cells. The mean percentage of WSX-1 was 70% in SK-MES, 66% in Calu-1, 97% in Calu-6, 77.5% in GLC82 and 78% in A549 cells. Experiments were performed in triplicate. **(C)** Regulation of angiogenesis-related gene expression in SK-MES (black bars) and Calu-6 (white bars) cells upon hrIL-27 treatment, as assessed by PCR Array. Histogram represents fold differences in gene expression of individual mRNA between cells cultured in the presence or absence of hrIL-27. Pooled results ± SD from two experiments performed in duplicate are shown. Regulation of stemness-related gene expression in SK-MES cells **(D)** and in Calu-6 cells **(E)** upon hrIL-27 treatment, as assessed by real-time RT-PCR. **(F)** Regulation of EMT-related gene expression in SK-MES cells upon hrIL-27 treatment. Results are representative of three independent experiments. *P < 0.05.

Expression of both chains of the IL-27R, gp130 and WSX-1 (TCCR, IL-27Rα), was investigated, by flow cytometry, in a series of cell lines derived from human lung AC, namely A549, GLC82, Calu-6 or from SCC, namely Calu-1 and SK-MES. As shown in Figure 1A and 1B, Calu-6 and SK-MES lines expressed the highest levels of both chains (gp130: 68% and 65%; WSX-1: 97% and 70% respectively) and were therefore chosen as representative of AC and SCC histotypes for the subsequent experiments.

We began by determining whether IL-27 affects the *in vitro* proliferation or apoptosis of these lines by culturing them with or without human (h) recombinant (r) hrIL-27 for 120 hours, and harvesting an aliquot every 24 hours to be analyzed for CFSE intracellular staining and for apoptosis. In both lines hrIL-27 was unable to directly modulate proliferation or apoptosis (not shown).

We next investigated whether IL-27 regulated, in both lines, sets of genes shaping tumor malignancy and specifically related to angiogenesis, stemness and invasiveness.

IL-27's ability to modulate angiogenesis-related genes in different cancer cell types, leading to anti-angiogenic effects *in vivo*, has been documented by us and others [8–10]. Unexpectedly, in both Calu-6 and SK-MES lines, hrIL-27 treatment considerably (*P* < 0.05) up-regulated, 18.53 and, 13.7 times respectively, the mRNA expression of *CXCL3* (Figure 1C), also known as Growth-Related Oncogene *(GRO)3*, GRO protein gamma *(GROγ)* and Macrophage Inflammatory Protein 2 beta *(MIP2β)*, an angiogenic ELR^+^ CXC chemokine [14], identified as a powerful neutrophil chemoattractant [15, 16], and also driving migration and adhesion of monocytes and macrophages [17].

Other angiogenesis-related genes were differently regulated by IL-27 in the two lines (Figure 1C). In SK-MES cells only, hrIL-27 up-regulated *IFNγ* (6.13 times) and down-regulated *LAMA5*, encoding for Laminin-α5 (4.5 times), and *THBS1*, encoding for Thrombospondin-1 (8.64 times), whereas in Calu-6 cells, hrIL-27 selectively up regulated Cadherin 5 (*CDH5*), i.e.: *Vascular Endothelial (VE)-Cadherin* (4.5 times), and the Tissue Inhibitor of Metalloproteinase-1 (*TYMP-1*) (5.3 times) encoding for Platelet Derived Endothelial Cell Growth Factor 1 (*ECGF1*).

Within a range of stemness-related genes (including *NANOG*, *BMI1*, *CD44v6*, *c-MYC*, see Supplementary information), in SK-MES cells, hrIL-27 significantly (*P* < 0.05) down-regulated mRNA expression levels of Octamer-binding Transcription Factor 4A, *OCT4A* (5.7 times), SRY (sex determining region Y)-box 2, *SOX2* (4.0 times), *SOX9* (7.1 times), Notch homolog 1, *NOTCH1* (7.0 times), and Krüppel-like factor 4, *KLF4* (6.1 times) (Figure 1D), whereas in Calu-6 cells it only down-modulated mRNA expression of *Sonic Hedgehog*, *SHH* (4.2 times) (Figure 1E). Moreover, in SK-MES cells, hrIL-27 down-modulated mRNA expression levels of *Nestin* (6.7 times), associated with cell stemness and EMT [18, 19], and within the *ZEB*, *SNAIL* and *TWIST* families of EMT-activating transcription factors [20, 21], hrIL-27 down-modulated mRNA expression of Snail family zinc finger 1, *SNAI1/SNAIL* (5.0 times), Snail family zinc finger 2, *SNAI2/SLUG* (4.3 times), and Zinc finger E-box binding homeobox 1, *ZEB1* (5.0 times) (Figure 1F), whereas the expression of *c-MET*, also involved in EMT [22, 23], and that of *ZEB2*, *TWIST1*, and *TWIST2* remained unaffected.

### IL-27 hinders tumour growth in pre-clinical xenograft models of lung cancer in association with a remarkable colliquative necrosis and apoptotic events

**Figure 2:**
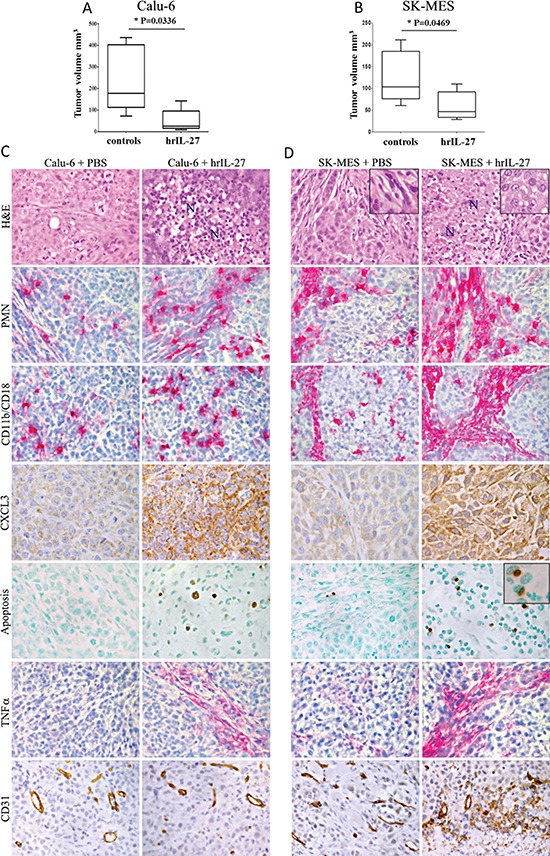
Antitumor Effects of IL-27 in Immunodeficient Pre-Clinical Models of Lung AC and SCC and Histopathological Analyses of the Tumor Growth or Regression Areas Volume of tumor masses developed in SCID/NOD **(A)** and athymic nude mice **(B)** injected with the Calu-6 and SK-MES cell lines respectively, and subsequently treated with PBS or hrIL-27. Results are representative of three independent experiments. *P < 0.05. Histological and immunohistochemical analyses **(C, D)** revealed that Calu-6 and SK-MES tumors grown in PBS treated mice had the features of poorly-differentiated human lung AC (rare glandular lumen) and SSC (epithelial cells with spindle morphology, inset), and displayed frequent mitotic figures, whereas both tumors from hrIL-27-treated mice showed wide areas of colliquative necrosis (N) and, particularly for SK-MES tumor cells, the acquisition of a more polygonal-round morphology (inset). Both histotypes from hrIL-27-treated mice showed a prominent granulocyte (PMN) and macrophage (CD11b/CD18) infiltrate, along with a strong CXCL3 production by tumor cells in comparison with control tumors. Following hrIL-27 treatment apoptotic events, close to cells endowed with segmented nuclei (inset), were frequent, as assessed by the TUNEL assay, in both histotypes, whereas they were almost absent in control tumors, and a distinct to strong expression of TNFα was detected at the sites of reactive infiltrates, whereas it was lacking in control tumors. The microvascular network supplying Calu-6 tumors from hrIL-27-treated mice was similar to that of control tumors. By contrast, in SK-MES tumors from hrIL-27-treated mice it was evidently impoverished, in comparison with control tumors. (C and D: H&E, PMN, CD11b/CD18, TNFα, and CD31 at X400; C and D: CXCL3, Apoptosis, and H&E insets at X630; D: Apoptosis inset: X1000).

*In vivo* studies using pre-clinical models of severe combined immunodeficient SCID/NOD and T-cell deficient athymic-nude mice, s.c. injected with Calu-6 and SK-MES cell lines respectively, showed that hrIL-27 considerably reduced tumor growth in both models. In particular, the mean tumor volume (mtv) ± standard error (SE) of Calu-6 tumors grown in hrIL-27-treated mice was 54.22 ± 24.2 mm^3^ *versus* 241 ± 69 mm^3^ of tumors from controls (*P* = 0.0336) (Figure 2A), whereas the mtv ± SE of SK-MES tumors was 59.08 ± 13.1 mm^3^ *versus* 123.3 ± 24.5 mm^3^ of tumors from controls (*P* = 0.0469) (Figure 2B).

To get an insight into the mechanisms underlying the *in vivo* antitumor effects of IL-27, tumor growth/suppression areas were histopathologically analyzed. The histologic features of Calu-6 and SK-MES tumors from control mice, recalled human poorly-differentiated AC and SSC, respectively. Tumors harvested from hrIL-27-treated mice displayed wide areas of colliquative necrosis characterized by a prominent reactive cell infiltrate, (Figure 2C and 2D), in addition, SK-MES tumors also presented evident alterations in cancer cell morphology ranging from a spindle to a polygonal-round phenotype (Figure 2D). Inflammatory infiltrates were wider in both Calu-6 and SK-MES tumors from hrIL-27-treated mice than in control tumors, because of the significant (*P* < 0.05) increase in their granulocyte and macrophage content, along with cancer cell expression of CXCL3 (Figure 2C and 2D) (Table 3). In addition to the areas of colliquative necrosis, frequent apoptotic features were evidenced, by the TUNEL assay, close to granulocytes identified by their segmented nuclei, (Figure 2C and 2D), in both Calu-6 and SK-MES tumors from hrIL-27-treated mice (Figure 2C and 2D) (Table 3).

To understand the molecular mechanism underlying apoptotic events *in vivo*, we next assessed in tumors from hrIL-27-treated and control mice the expression of apoptosis-inducing proteins Tumor Necrosis Factor (TNF)α and TNF-Related Apoptosis Inducing Ligand (TRAIL). Immunostainings revealed that TRAIL was almost undetectable, while TNFα expression was distinct to strong, in the foci of reactive infiltrates, in both tumor types harvested from hrIL-27-treated mice (Figure 2C and 2D) (Table 3).

**Figure 3:**
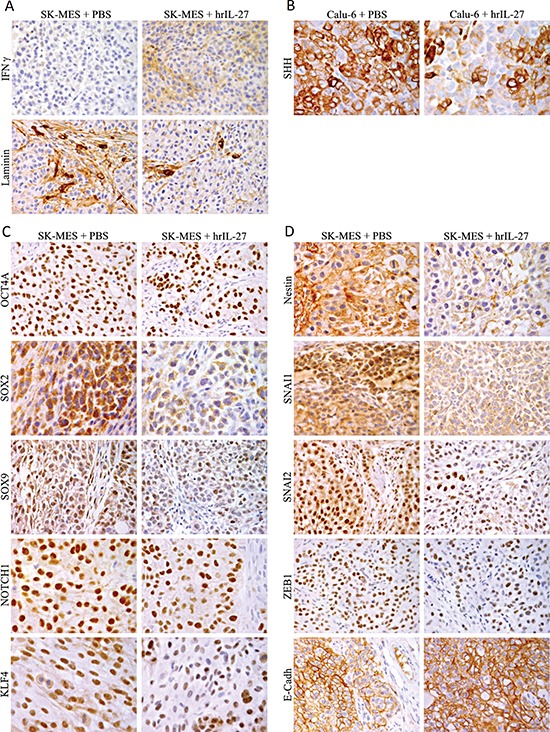
Expression of IFNγ and Down-Modulation of Stemness Genes and EMT-Regulating Transcription Factors by IL-27 in SK-MES Tumors *in vivo* **(A)** An evident weakness of the laminin network and a slight, but distinct expression of IFNγ were observed in SK-MES tumors harvested from hrIL-27 treated mice in comparison with control tumors. (X400). **(B)** In comparison with Calu-6 tumors from control mice, those from hrIL-27-treated mice presented a reduced percentage of cancer cells endowed with a distinct to strong expression of SHH. (X630). **(C)** In comparison with SK-MES tumors from control mice, those from hrIL-27-treated mice showed a reduced percentage of cancer cells endowed with a distinct to strong expression of OCT4A, SOX2, SOX9, NOTCH1, and KLF4. (OCT4A and SOX9: X400; SOX2, NOTCH1, and KLF4: X630). **(D)** Expression of Nestin was faint following hrIL-27 treatment, while it was distinct to strong in control tumors. Nuclear expression of SNAI1, SNAI2, and ZEB1 was less frequent in tumors from hrIL-27 treated mice than in control tumors, and the tumor cell surface expression of E-Cadherin was stronger in the former than in the latter. (Nestin: X630; SNAI1, SNAI2, ZEB1, and E-Cadherin: X400).

Lastly immunohistochemical analyses revealed that the microvascular network of Calu-6 tumors from hrIL-27-treated mice was similar to that of the controls whereas that of SK-MES tumors from hrIL-27-treated mice was clearly impaired (Figure 2C and 2D) (Table 3) in association with a weakened laminin network and a faint, but distinct cancer cell expression of IFNγ (Figure 3A).

### IL-27 down-modulates stemness- and EMT-related genes, particularly in SCC tumors

To assess whether IL-27 regulation of pluripotency- and EMT-related genes also occurs *in vivo* at protein level, we carried out immunohistochemical analyses of tumors from hrIL-27- and PBS-treated mice.

In Calu-6 tumors from hrIL-27-treated animals, the percentage of cancer cells displaying a distinct to strong SHH staining was decreased compared with control tumors (Figure 3B), whereas in SK-MES tumors from treated animals, the percentage of cells endowed with a distinct to strong nuclear staining for OCT4A, NOTCH1, and KLF4 was clearly decreased (Table 3) as was reduced the percentage of tumor cells displaying both nuclear and cytoplasmic SOX2 positivity and nuclear SOX9 positivity (Figure 3C) (Table 3). Expression of Nestin was weakened in cancer cells forming SK-MES tumors from IL-27-treated mice (Figure 3D). Nuclear and cytoplasmic SNAI1 stainings were dramatically and moderately reduced respectively, and the percentage of cancer cells endowed with a distinct to strong nuclear SNAI1, SNAI2, and ZEB1 staining also significantly (*P* < 0.05) decreased following hrIL-27 treatment (Figure 3D), whereas E-Cadherin expression was reinforced in tumors from hrIL27-treated mice (Figure 3D) (Table 3).

### Myeloablation by treosulfan thwarts the *in vivo* anti-lung cancer effects of IL-27

To assess whether granulocytes and macrophages may, as suggested by the morphological data, account for the anti-lung cancer effects of IL-27 *in vivo*, we next repeated tumor growth experiments in mice pre-treated with myeloablative doses of treosulfan to obtain a severe or complete depletion of bone marrow cells [18].

**Figure 4:**
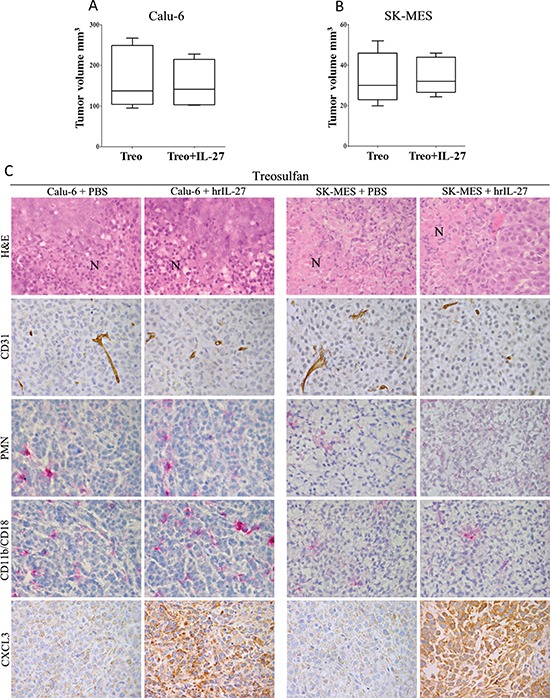
Loss of IL-27 Anti-Lung Cancer Effects in Myeloablated Mice and Histopathological Features of their Tumors The mean volume of Calu-6 **(A)** and SK-MES **(B)** tumors grown in myeloablated mice treated with hrIL-27 was not significantly different (Calu-6: *P* = 0.0317 and SK-MES: *P* = 0.0159) from that of tumors grown in myeloablated PBS-treated mice. Results are representative of three independent experiments. **(C)** Histopathological analyses revealed that both Calu-6 and SK-MES tumors from hrIL-27-treated and myeloablated mice developed small foci of ischaemic necrosis (N) as their respective control tumors, in association with a rarefied vascularization. Tumor infiltrating granulocytes and macrophages were barely detected in both histotypes from IL-27-treated and PBS-treated, myeloablated mice, in spite of the distinct CXCL3 production found in both histotypes from hrIL-27-treated mice in comparison with control tumors. (X400).

These experiments revealed that after this treatment both Calu-6 and SK-MES tumors grew similarly in hrIL-27-treated and control mice. In particular, the mtv ± SE of Calu-6 tumors harvested from myeloablated and IL-27-treated mice was 153.4 ± 29.9 mm^3^ *versus* 169 ± 33.99 mm^3^ of tumors from myeloablated controls (Figure 4A). The mtv ± SE of SK-MES tumors from myeloablated and IL-27-treated mice was 34.6 ± 4 mm^3^ *versus* 33.6 ± 5.65 mm^3^ of tumors from myeloablated controls (Figure 4B). Of note, both Calu-6 and, particularly, SK-MES tumors grown in myeloablated control mice were smaller than tumors developed in not-myeloablated control mice (Calu-6: *P* = 0.0317 and SK-MES: *P* = 0.0159).

Histological features of both Calu-6 and SK-MES tumors developed in myeloablated hrIL-27-treated mice were similar to those from myeloablated controls. However, both histotypes, developed in myeloablated mice, independently from hrIL-27-treatment, presented small ischaemic necrotic foci (Figure 4C) in association with an evident (vessel counts: 3 ± 2 in Calu-6, and 5 ± 2 in SK-MES tumors from myeloablated controls, *versus* 9 ± 3 in Calu-6, and 12 ± 4 in SK-MES tumors from non-myeloablated controls; *P* < 0.05) decrease of the whole vascular supply (Figure 4C). These tumours were almost devoid of granulocyte and macrophage content (Figure 4C). CXCL3 was still firmly expressed by tumor cells in both Calu-6 and SK-MES tumors from myeloablated and hrIL-27-treated mice (Figure 4C), while a faint IFNγ production was only detected in the latter.

### Human lung AC and SCC, and their precursor lesions express IL-27R in neoplastic and dysplastic cells, microvessels and tumor-associated reactive cells

**Figure 5:**
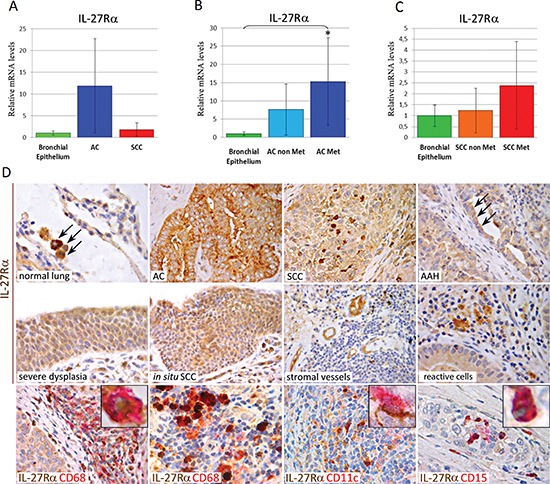
mRNA and Protein Expression of IL-27Rα in Human Normal Lung, Pre-Neoplastic Lesions, Lung AC and SCC **(A, B and C)** Histograms representing the relative expression ± SD of IL-27Rα mRNA in microdissected neoplastic cell populations from groups of 10/12 samples of each cancer subtype: AC and SCC, in **(A)** or metastatic (Met) *versus* non-metastatic (non Met) primary AC **(B)**, or Met *versus* non Met primary SCC **(C)** compared with histologically normal bronchial epithelium from the same patients, normalized with the housekeeping gene HPRT. One-way ANOVA for comparisons between normal bronchial epithelium, AC and SCC (A) (*P* = 1.0), or between normal bronchial epithelium, non-Met AC and Met AC (B) (*P* = 0.045, *P < 0.05 Tukey HSD Test compared with normal bronchial epithelium), or between normal bronchial epithelium, non-Met SCC and Met SCC (C) (*P* = 0.190). **(D)** Immunohistochemistry revealed that IL-27Rα (*brown*) was expressed in monocyte/macrophage-like cells in the normal lung tissue (X630), in most of AC (X400) and SCC (X630) and frequently in dysplastic cells forming AAH (arrows) (X630), severe dysplasia (X630) and *in situ* SCC (X400). It may also be found in stromal small vessels (X400) and infiltrating reactive cells (X630), particularly in CD68^+^ macrophages (*red*) (X630, inset at X1000) and CD11c^+^ myeloid dendritic cells (*red*) (X630, inset at X1000) scattered in the stroma (in the bottom left panel) or intermingled with lymphocytes in TLS (in the bottom middle panels). CD15^+^ granulocytes (*red*) (in the bottom right panel), which may be found in the small necrotic foci of rapidly developing SCC, also express IL-27Rα (*brown dots*) (X630, inset at X1000).

To determine whether lung cancer patients could benefit from IL-27's antitumor effects, we next immunohistochemically evaluated the expression and distribution of IL-27R in AC and SCC tissue sections. Since expression of gp130 has been documented in lung cancer [24, 25], we only assessed IL-27Rα expression in both cancerous and normal lung samples (from both cancer and control patients). In the normal tissue, it was basically found in mononuclear/macrophage-like cells fluctuating within alveolar walls (Figure 5D). Normal bronchial epithelia firmly expressed *IL-27Rα* mRNA, whereas in neoplastic samples it was expressed by the majority of AC, 90%, and SCC, 84% (Table 2) (Figure 5A and 5D). Furthermore, within AC, metastatic tumors revealed significantly (*P* < 0.05) higher expression levels of *IL-27Rα* mRNA than normal bronchial epithelia (Figure 5B). Notably, *IL-27Rα* expression in microdissected bronchial epithelium from normal samples of patients with lung cancer was analogous to that in control patients. These molecular data showed a good correlation (ρ = 0.82) with the immunohistochemical findings.

IL-27Rα expression by the primary tumor was significantly associated with lymph node status (*P* = 0,001), and advanced stages of disease (*P* = 0,02) as assessed by Fisher's exact test, whereas no significant association was observed with patient age or smoking history.

Immunohistochemistry also revealed IL-27Rα expression in AC and SCC precursor lesions, namely atypical adenomatous hyperplasia (AAH) and severe dysplasia, squamous metaplasia (SM), squamous cell carcinoma *in situ* (SCIS) (Figure 5D), respectively (Table 1). IL-27Rα expression may also be found in microvessels and infiltrating immune cells (Figure 5C), mostly identifiable as CD68^+^ monocytes/macrophages (Figure 5D) and CD11c^+^ myeloid dendritic cells. They were found in the stroma of both AC and SCC, scattered or within the lymph node–like structures, known as tertiary lymphoid structures (TLS) (Figure 5D) [26, 27]. CD15^+^ granulocytes that may be found in necrotic foci of rapidly growing tumors also expressed IL-27Rα (Figure 5D).

**Table 1:** Clinic-pathological characteristics of patients with premalignant lung lesions and IL-27Rα expression profiles of these lesions

Variables		IL-27Rα immunostaining*		
		Positive	Weakly Positive	Negative
***Age:*** (range 50–78)				
***Gender***				
Male	14	10	3	—
Female	6	5	1	—
***Histological type***				
AAH	7	7	—	—
SM and Dysplasia (mild, moderate, severe)	7	4	2	—
SCIS	6	4	2	—

* IL-27Rα immunostaining was scored as negative, positive or weakly positive as described in Methods.

## DISCUSSION

AC and SCC constitute the commonly diagnosed lung cancer histotypes [1, 2], but their management still results in low overall cure rates, suggesting the need for novel therapeutic approaches. The idea of strengthening patient's immune system to fight cancer is of growing interest for oncologists [28]. Our data indicate that IL-27, a well-tolerated and toxicity-free cytokine [9] may provide a new therapeutic option in NSCLC. Previous studies assessing IL-27's effects in lung cancer have used mouse autograft models [11, 13] or, *in vitro* experiments with both murine and human lung AC cell lines [11, 12]. Our data confirm, in pre-clinical xenograft models of human lung AC and SCC, the anti-lung cancer effects of IL-27. They have also revealed unforeseen implications for its immunological antitumor capability in the form of I. boosting the potent granulocyte/macrophage chemo-attractant CXCL3, in both AC and SCC cells, leading to intratumoral myeloid cell recruitment and activation, and II. re-education of these immune cells from the status of “cancer feeder”, and host-detrimental [29, 30] towards that of “cancer killer”, thus host-beneficial [30–32].

Tumor destruction by the prominent neutrophil and macrophage influx mediated by *CXCL3* [15–17] overcomes, at least in our setting, its well-known pro-angiogenic effects [14], since microvessel density remained unaltered in AC and slightly decreased in SCC tumors from IL-27-treated animals, when compared with controls. Anti-angiogenesis has a marginal or nil role in IL-27's anti-lung cancer efficacy, and apart from a slight *IFNγ* induction and *LAMININ-α5* down-regulation observed in SCC cells, IL-27 even down-regulates the angiogenesis inhibitor *THROMBOSPONDIN-1* [33] in SK-MES cells, and up-regulates *VE-CADHERIN* [34] and the pro-angiogenesis gene *TYMP-1*, encoding for *ECGF1* [35], in Calu-6 cells, but without significant *in vivo* consequences for tumor vascularity.

Granulocytes and macrophages are endowed with IL-27R, and may respond to IL-27 by increasing their oxidative burst and cytokine production [36–38], suggesting that the range of action for this cytokine is not restricted to T cells, the key mediators of its antitumor effect in an immune-intact host [9]. Our results provide the first evidence that intratumor recruited and activated myeloid cells may, in a B/T cell-deficient host, take the place of T lymphocytes in mediating IL-27's antitumor activity, leading to a dramatic colliquative necrosis, and TNFα-associated apoptotic events in both AC and SCC.

While extending to human SCC *in vivo* the finding of IL-27's down-modulation of EMT transcription factors such as SNAI1, SNAI2 and ZEB1 [20, 21], our discoveries identify a novel role for IL-27 as a negative regulator of pluripotency genes such as SHH in human AC cells, and SOX2, OCT4A, NOTCH1, KLF4, SOX9 and Nestin [18, 19] in human SCC cells, both *in vitro* and *in vivo*. EMT, a key event during the early phases of invasion and metastatization, selects for stem cell property [39], which, in turn, may condition the self-renewal capability of a cancer and correlate with its aggressiveness [40, 41]. Down-regulation of critical pluripotency genes by IL-27 increases the propensity of cells to differentiate towards a less aggressive phenotype [42–44] as shown by their transition from a fibroblast-like to a polygonal-round E-Cadherin-positive phenotype.

Tumor responsiveness to IL-27, however, is the prerequisite for its entry into clinical trials. Most AC and SCC express IL-27R, and those with N1 involvement are often endowed with higher expression levels. Dibra et al. observed that WSX-1 expression in tumors induces immune tolerance exerting pro-tumorigenic functions, independently from IL-27 [45]. Our data, drawn from patient samples, suggest that IL-27 may be used to overcome this drawback and be of particular benefit in advanced lung cancer stages. The myeloid cell mediated anti-lung cancer effects of IL-27 may be exerted in humans, since in our samples IL-27R is not only expressed by cancer cells, and microvessels, but also by CD15^+^ granulocytes, CD68^+^ monocytes/macrophages and CD11c^+^ myeloid dendritic cells scattered in the stroma or arranged in TLS. TLS have been associated with a favorable clinical outcome in NSCLC [26, 27] and IL-27 may promote, in these lymphoid structures, an efficient adaptive antitumor immunity.

Altogether, our results highlight novel aspects of IL-27's antitumor potential, specifically in NSCLC, such as the ability I. to drive myeloid cells towards antitumor activities, and II. down-regulate stemness genes, particularly in SCC cells, thus suppressing their self-renewal potential. IL-27 may thus be proposed for clinical trials with the prospect of its clinical use in immune-defective or advanced NSCLC patients.

## MATERIALS AND METHODS

### Ethics statement

Animal experiments were performed at the IRCCS “San Martino” National Institute for Cancer Research, Genoa, in keeping with the National and International regulations (Italian Legislative Order 27/01/1992, n.116, European Economic Community Council Directive 86/609, OJL 358, Dec. 1, 1987). For studies on human tissues, written informed consent was obtained from patients. The study was approved by the Ethical Committee of the “G. d'Annunzio” University of Chieti (Italy), and Local Health Authority No.2, in the report n.14 of 19/07/2012, and performed in accordance with the principles outlined in the Declaration of Helsinki.

### Patients and samples

Premalignant and malignant lung samples were obtained from 78 untreated patients with operable NSCLC staged IA–IIIA according to the pTNM system [46]. NSCLC consisted of 33 AC (12/33 with N1 involvement) and 25 SCC (10/25 with N1 involvement). Our panel of 20 premalignant and 58 malignant lesions [47, 48], and patient's clinic-pathological characteristics [48, 49] are shown in Tables 1 and 2. Normal lung tissue was obtained from both lung cancer patients and control patients operated for other reasons. Sample collection and processing are described in the Supplementary information.

**Table 2:** Clinic-pathological characteristics of patients with lung cancer and IL-27Rα expression profiles of these cancers

Variables		IL-27Rα immunostaining of the primary tumor*		
		Positive	Weakly positive	Negative
***Age*** (range 38–79)				
***Gender***				
Male	45	13 (29%)	26 (58%)	6 (13%)
Female	13	7 (54%)	5 (38%)	1 (8%)
***Histological type***				
*NSCLC*	*58*	*20 (34%)*	*31 (54%)*	*7 (12%)*
AC	33	15 (45%)	15 (45%)	3 (10%)
SCC	25	5 (20%)	16 (64%)	4 (16%)
***Tumor size***				
T1	29	8 (28%)	16 (55%)	5 (17%)
T2	27	12 (45%)	13 (48%)	2 (7%)
T3	2	2 (100%)	—	—
***Lymph node status***				
N0	34	6 (18%)	21 (62%)	7 (20%)
N1	24	14 (58%)	10 (42%)	—
N2	—	—	—	—
***Stage***				
IA	16	2 (13%)	9 (56%)	5 (31%)
IB	18	4 (22%)	12 (67%)	2 (11%)
IIA	13	8 (62%)	5 (38%)	—
IIB	9	4 (44%)	5 (56%)	—
IIIA	2	2 (100%)	—	—
***Smoking history***				
Smokers	42	16 (38%)	20 (48%)	6 (14%)
Non smokers	16	4 (25%)	11 (69%)	1 (6%)

* IL-27Rα immunostaining was scored as negative, positive or weakly positive as described in Methods.

**Table 3:** Immunohistochemical analyses of tumors developed after subcutaneous injection of Calu-6 or SK-MES cells in SCID/NOD and athymic NU/NU Mice, respectively, and treated with PBS or hrIL-27

	Calu-6						SK-MES					
	PBS			hrIL-27			PBS			hrIL-27		
**Immune Cells**												
Granulocytes	7.0	±	3.3	25.0	±	7.2^†^	14.5	±	5.6	31.4	±	6.0^†^
Macrophages	8.5	±	4.1	21.4	±	6.3^†^	12.0	±	4.2	27.0	±	6.5^†^
**Blood Vessels**	9.8	±	4.0	7.7	±	3.0	12.8	±	4.9	5.0	±	2.2^†^
**Apoptotic Index**	2.5	±	2.0%	8.9	±	3.2%^†^	3.4	±	2.2%	10.5	±	3.5%^†^
Human Cytokines*												
CXCL3		±			+ +			±			+ +	
IFNγ		─			─			─			+	
TNFα		─			+			±			+ +	
Nestin*		ND			ND			++			±	
E-Cadherin*		ND			ND			+			+ +	
**Stemness genes**												
SHH	88.0	±	8.9%	58.5	±	10.3%^†^						
OCT4A		ND^‡^			ND		85.0	±	11.5%	59.4	±	12.2%^†^
SOX2		ND			ND		79.2	±	9.0%	40.5	±	15.6%^†^
SOX9		ND			ND		84.5	±	11.3%	62.5	±	9.0%^†^
NOTCH1		ND			ND		86.2	±	8.0%	63.1	±	10.5%^†^
KLF4		ND			ND		78.3	±	10.0%	39.0	±	14.2%^†^
**EMT-related genes**												
SNAI1		ND			ND		85.3	±	9.0%	66.4	±	8.0%^†^
SNAI2		ND			ND		80.5	±	8.2%	58.5	±	10.3%^†^
ZEB1		ND			ND		71.8	±	10.0%	49.7	±	9.2%^†^

Assessment of cytokine, Nestin and E-cadherin expression, and counts of microvessels, immune cells, TUNEL positive cells, stemness and EMT-related gene expressing cells were performed at X400 in a 0.180 mm^2^ field. At least 3 samples (three sections/sample), and 6–8 (depending on the tumor width) randomly chosen fields/section were evaluated. Results are expressed as mean ± SD of CD31 positive microvessels per field; or RB6-8C5 (granulocytes) or CD11b/CD18 (macrophages) positive cells per field; or as mean ± SD percentage of TUNEL positive cells, or stemness- or EMT-related gene expressing cells evaluated on paraffin embedded sections by immunohistochemistry.

* The expression of cytokines, Nestin and E-Cadherin was defined as absent (─); scarce (±); distinct (+) or strong (++) on paraffin embedded (CXCL3, IFNγ, Nestin and E-Cadherin) or frozen (TNFα) sections stained with the corresponding Ab.

† Values significantly different (*P* < 0.05) from corresponding values in tumors developed in PBS-treated mice.

‡ ND, not detected.

### Cell culture and flow cytometry

Human lung SCC cell lines, Calu-1 and SK-MES, and AC cell lines, Calu-6, GLC82 and A549 (ATCC cell bank) were cultured in RPMI 1640 with 10% FCS (Seromed Biochrom KG, Berlin, Germany). HrIL-27 (R&D System, Abingdon, UK) was used at 100 ng/ml following titration experiments. Details on the assessment of IL-27R expression, tumor cell proliferation and apoptosis by flow cytometry are provided in the Supplementary information.

### Assessment of angiogenesis, stemness- and EMT-related gene expression by real-time RT-PCR

RNA was extracted from Calu-6 and SK-MES cells cultured for 24 hours in the presence or absence of hrIL-27, using TRIZOL reagent (Invitrogen, Paisley, UK). Contaminant genomic DNA was removed by DNase treatment (Qiagen GmbH, Hilden, D). RNA was reverse-transcribed with the RT^2^ First Strand cDNA Synthesis kit (SABioscience, Frederick, MD, USA). Experiments are described in the Supplementary information.

### Laser capture microdissection (LCM) and real-time RT-PCR

We used the P.A.L.M. Micro Beam System (Bernried, Germany) for LCM of frozen sections from normal human lung, AC, and SCC samples. We selected the bronchial epithelium from normal lung sections and tumor cells from AC or SCC sections. Details are provided in the Supplementary information.

### Mouse studies

Four- to six-week-old athymic-nude and SCID/NOD mice (Harlan Laboratories, Udine, Italy) were housed under specific pathogen-free conditions. Details on Tumor growth experiments in mice pre-treated or not with the myeloablative agent treosulfan (Medac) [50], and schedule of hrIL-27 administration are provided in the Supplementary information.

### Histopathological and morphometric analyses

Methods for histology, immunohistochemistry, TUNEL assay, and the list of Abs used are reported in the Supplementary information.

IL-27Rα expression by pre-neoplastic and neoplastic lesions was defined as positive, weakly positive, or negative. Details on the evaluation of IL-27Rα expression, in human samples, and of Nestin, E-Cadherin, and cytokine expression; counts of microvessels, and immune cells, percentage of apoptotic cells, and cancer cells expressing stemness or EMT-related genes, in tumor xenografts, are reported in the Supplementary information.

### Statistical analysis

Tumor volumes were reported in mm^3^ *versus* time. Differences in tumor volume, microvessel density and counts of immune cells or percentage of apoptotic cells or stemness and EMT-gene expressing cells between tumors from hrIL-27- and PBS-treated mice were assessed by Student's *t* test, and data were reported as mean ± standard deviation (SD). Between-group differences in the relative expression of IL-27Rα mRNA, by real-time RT-PCR, were assessed by one-way analysis of variance (ANOVA) and the difference between each pair of means was evaluated with the Tukey's HSD test.

Fisher's exact test was used to examine the association between IL-27Rα protein expression, evaluated by immunohistochemistry, in primary lung tumors, and clinic-pathological characteristics. The Spearman rank correlation coefficient (ρ) was used to examine the correlation between immunohistochemical staining and real-time RT-PCR for IL-27Rα expression. The SPSS software, version 11.0 (IBM, Armonk, NY, USA) was employed, with *P* < 0.05 as the significance cut-off.

## SUPPLEMENTARY INFORMATION



## Abbreviations

AAH: Atypical Adenomatous Hyperplasia
AC: Adenocarcinoma
BMI1: Polycomb complex protein 1
CDH: Cadherin
CXCL3: Chemokine (CXC motif) ligand 3
ECGF1: Platelet-Derived Endothelial Cell Growth Factor 1
EMT: Epithelial-Mesenchymal Transition
GROγ: Growth Related Oncogene protein gamma
IFNγ: Interferon gamma
IL: Interleukin
KLF4: Krüppel-Like Factor 4
LCM: Laser Capture Microdissection
LLC1: Lewis Lung Carcinoma line 1
MIP2β: Macrophage Inflammatory Protein 2 beta
mtv: mean tumor volume
NANOG: Nanog Homeobox
NOTCH1: Notch homolog 1
NSCLC: Non-Small Cell Lung Cancer
OCT4A: Octamer-Binding Transcription factor 4 A
R: Receptor
SCC: Squamous Cell Carcinoma
SCIS: squamous cell carcinoma *in situ*
SE: standard error
SHH: Sonic Hedgehog
SM: Squamous Metaplasia
SOX: SRY (sex determining region Y)-box
TYMP-1: Tissue Inhibitor of Metalloproteinase-1
TLS: Tertiary Lymphoid Structures
TNF: Tumor Necrosis Factor
TRAIL: TNF-Related Apoptosis Inducing Ligand
TUNEL: Terminal deoxynucleotidyl transferase dUTP nick end labeling
TWIST: twist family bHLH transcription factor
VE: Vascular Endothelial
ZEB: Zinc finger E-box binding homeobox.
