# Supplementary material for: Interleukin-27 re-educates intratumoral myeloid cells and down-regulates stemness genes in non-small cell lung cancer
Source: Oncotarget. 2015 Jan 3;6(6):3694–708. doi: 10.18632/oncotarget.2797 (PMC4414147; doi:10.18632/oncotarget.2797)
Supplement: Supplementary file 1 [file oncotarget-06-3694-s001.pdf]

SUPPLEMENTARY INFORMATION

Patients and samples

Normal lung tissue was obtained from both lung cancer patients and control patients operated for other reasons. Macroscopically normal lung samples, taken not less than 2 cm from the tumor or other lung lesions, were confirmed as histologically normal. One half of each normal or neoplastic sample was fixed in 4% formalin and embedded in paraffin, and the other was embedded in Killik frozen section medium (Bio-Optica, Milano, Italy) snap-frozen in liquid nitrogen, within 10 min from excision, and preserved at –80°C.

Cell proliferation and apoptosis

The expression of both chains of IL-27R was analyzed using FITC conjugated anti-gp130 and PE-conjugated anti-WSX-1(IL-27Rα) mAbs (both from R&D Systems). Isotype-matched antibodies of irrelevant specificity (Caltag, Burlingame, CA, USA) were used as controls. Cells were run on Gallios flow cytometer, acquiring at least 10^4 events, and data were analyzed using Kaluza analysis software (Beckman Coulter). The human Calu-6 and SK-MES lung carcinoma cell lines were cultured for 24, 48, 72, 96 and 120 hours with or without 100 ng/ml hrIL-27. Cells were incubated with 2 μM Carboxy-Fluorescein diacetate Succinimudyl Ester (CFSE) in RPMI 1% FCS for 15 minutes at 37°C, washed in RPMI 10% FCS, plated and analyzed by flow cytometry at the above mentioned time points. Apoptosis was assessed using the Annexin V-FITC Kit from Immunostep, and apoptotic cells were identified as Annexin V+/PI+ cells by flow cytometry.

Mouse studies

Two groups of 10 athymic-nude animals were injected sub-cutaneously (s.c.) with $4x10^6$ SK- MES and two groups of 8 SCID/NOD mice were injected s.c. with $6x10^6$ Calu-6 cells. One group of 10 athymic-nude mice and 8 SCID/NOD mice were treated s.c. with 2 weekly doses of hrIL-27 (1μg/mouse/dose) starting from 2 days after tumor cell injection. The other group of SCID/NOD and of athymic-nude mice was injected with PBS (controls) according to the same schedule. Mice were sacrificed at day fourteen when signs of poor health were evident. Their tumors were removed, measured with a caliper, formalin-fixed or frozen in O.C.T. compound, and subjected to histopathological analyses.

Tumor growth experiments were then repeated in mice myeloablated by treosulfan (Medac). Twelve SCID/NOD and 12 nude mice were pre-treated intra-peritoneally for three consecutive days with treosulfan (1500 mg/Kg/day, Medac) that is a myeloablative agent. The day after the last treatment, mice were injected with Calu-6 or SK-MES cells as described for experiments performed in non-myeloablated mice. Six animals from each group were then treated with hrIL-27, the other six with PBS. Animals injected with SK-MES cells were sacrificed at day +14 from tumor cell inoculation, whereas animal injected with Calu-6 at day +19. Tumors were removed, measured, formalin-fixed or frozen in O.C.T. compound, and subjected to morphological and immunohistochemical analyses.

Histology, immunohistochemistry and morphometric analyses

For histology, paraffin-embedded samples were sectioned at 3 μm and stained with hematoxylin and eosin (H&E). Single immunohistochemistry was done on paraffin-embedded or frozen sections, depending on the antibody (Ab) used, whereas double immunohistochemistry was done on paraffin-embedded sections.

For immunohistochemistry on the formalin-fixed, paraffin-embedded samples, sections were deparaffinized and, after antigen retrieval, incubated for 30 minutes with primary antibodies listed in Table S1. Immune complexes were detected using the Bond Polymer Refine Detection Kit according to the manufacturer's protocol (Leica Biosystems, Wetzlar, Germany) or using the avidin-biotin complex method, depending on Ab host species.

For immunohistochemistry on frozen samples, cryostat sections were fixed in acetone for 10 minutes and, after washing in PBS/Tween-20, incubated with primary Abs listed in Table S1. Immune complexes were detected using the avidin-biotin complex method, then sections were counterstained with hematoxylin.

For WSX-1/CD68, WSX-1/CD15 and WSX-1/CD11c double stainings on formalin-fixed paraffin-embedded samples, sections were deparaffinized, subjected to antigen retrieval, treated with H_2O_2/3% for 5 minutes to inhibit endogenous peroxidase, and then washed in H_2O. The slices were then incubated for 30 minutes with the first primary Ab (anti-WSX-1) followed by detection with the Bond Polymer Refine Detection Kit (Leica Biosystems) according to the manufacturer's protocol. Then, sections were incubated for 30 minutes with the second primary Ab (anti-CD68, anti-CD15 and anti-CD11c) followed by detection with the Bond Polymer Refine Red Detection Kit (Leica Biosystems) according to the manufacturer's protocol.

WSX-1 (IL27Rα) expression by preneoplastic lesions and tumors was evaluated using the following criteria based on 1) the widening of the staining expressed as the

percentage of tumor stained i.e.: < 50%, ≥ 50% ≤ 70%, and > 70%, and 2) the strength of the staining: defined as absent (–), scarce (±), moderate (+) or strong (+ +).

Thus, immunostaining was defined as **positive** when a) the widening was > 70% and its strength range scarce (±) to strong (+ +), or b) the widening was > 50% ≤ 70% and its strength range moderate (+) to strong (+ +); **weakly positive** when a) the widening was > 50% ≤ 70% and its strength was scarce (±) or b) the widening was = 50% and its strength range scarce (±) to strong (+ +); **negative** when the widening was ≤ 50% and its strength was scarce (±) to absent (–).

Immunostained sections were examined by two pathologists with very good agreement (κ value = 0.78 and 0.83, for evaluations of immunostained tissue sections, according to reference [S1].

Hematic microvessels were identified as small tubes or circles marked by anti-CD31 Ab. Assessment of cytokine expression and counts of microvessels, immune cells and apoptotic cells were performed at X400 in a 0.180 mm^2 field. At least 3 samples (1 sample / tumor growth area) and 6–8 (depending on the tumor width) randomly chosen fields/sample were evaluated.

Results are expressed as mean ± SD of CD31 positive microvessels per field; or RB6-8C5 or CD11b/CD18 positive cells per field; or TUNEL positive cells/number of total cells evaluated on formalin-fixed (CD31, TUNEL) or frozen (RB6-8C5, CD11b/CD18) sections by immunohistochemistry.

The expression of cytokines, Nestin and E-cadherin in tumor xenograft was defined as absent (−), scanty (±), distinct (+) or strong (+ +) on paraffin embedded (CXCL3, IFNγ, Nestin, E- Cadherin) or frozen (TNFα) sections stained with the corresponding Ab.

Expression of stemness- (SHH, OCT4A, SOX2, SOX9, NOTCH1, and KLF4) or EMT- (SNAI1, SNAI2, and ZEB1) related genes, in cancer cells forming tumor xenograft, was defined as absent (−), weak (±), distinct (+), or strong (+ +). The percentage of cancer cells expressing stemness- or EMT-related genes, in tumor xenograft, was measured by quantifying the fraction of cells endowed with a distinct (+) to strong (+ +) expression/number of total cells, in immunostained tissue sections. Six to 8 high-power fields were examined per section and three sections per sample were evaluated. Results were expressed as the mean percentage ± SD of positive cells/number of total cells evaluated by light microscopy on single immunostained sections at x400 in a 0.180 mm^2 field.

Terminal deoxynucleotidyl transferase–mediated dutp nick end labeling (TUNEL) assay

DNA fragmentation associated with apoptosis was detected in 4 µm sections by TUNEL staining with the ApopTag plus Peroxidase in Situ Apoptosis kit (Millipore, Billerica, MA, USA) according to the manufacturer's protocol.

Laser capture microdissection (LCM) of human lung tissue samples

For LCM, we used the P.A.L.M. Micro Beam System (P.A.L.M. Microlaser Technologies, Bernried, Germany). 10 µm frozen sections from normal lung, AC and SCC sample were mounted on PEN-membrane covered slides (P.A.L.M.), thawed at room temperature, and immersed in cold acetone (5 minutes). Two sections per sample were microdissected. All reagents were prepared using Ultrapure DNAse/RNAse-Free distilled water (Invitrogen, Paisley, UK). Immediately after H&E staining, sections were used for LCM.

We selected the bronchial epithelium from normal lung sections and tumor cells from AC or SCC sections. Selected cells were cut and catapulted intact into the cap of a LPC-Microfuge Tube (P.A.L.M.), and RNA was immediately isolated with the RNeasy micro kit (Qiagen, Hilden, Germany) according to the manufacturer's protocol.

Real-time RT-PCR

Human Angiogenesis RT^2 PCR Array and RT^2 Real-Time SYBR Green/ROX PCR Mix were from SABioscience. PCR was performed on ABI Prism 7500 Sequence Detector (Applied Biosystems). Results were normalized on the median value of a set of housekeeping genes. Changes in gene expression between hrIL-27 treated and control samples were calculated using the DDCt method. Results from three different hrIL-27 treated and control samples were pooled and analyzed by software provided by the manufacturer. A significant threshold of four-fold change in gene expression corresponded to $P < 0.001$.

The real-time RT-PCR was performed on RNA extracted from microdissected cells, to assess expression levels of *WSX-1*, and from hrIL-27 treated and untreated Calu-6 and SK-MES cell lines, to assess expression levels of stemness- and EMT-regulating transcription factors, using the QuantiTect Reverse Transcription Kit for the reverse transcription and the QuantiFast SYBR Green PCR Kit for real-time PCR (both from Qiagen).

The primers for *NANOG, SNAI1, SNAI2, SOX2, TWIST1, TWIST2, ZEB1, ZEB2,* and the housekeeping gene hypoxanthine phosphoribosyltransferase 1 (*HPRT*) were designed with Beacon Designer software (Premier Biosoft International, Palo Alto, CA, USA) in our laboratory, whereas the primers for *CD44v6* and *OCT4A* were designed as reported[S2, S3], and all of them synthesized by Sigma-Aldrich Corporation (St. Louis, MO, USA): *NANOG* forward 5′-TCTTCCACCAGTCCCAAA-3′ and *NANOG* reverse 5′- GCGTCACACCATTGCTAT-3′; *Nestin* forward 5′-CGTCGGTCTCTTTTCTCTTC-3′ and *Nestin* reverse 5′-GCTCCCACATCTGAAACG-3′; *SNAI1*

forward 5′- CCTCTTCCTCTCCATACCT-3′ and *SNAI1* reverse 5′-TTCATCAAAGTCCTGTGGG-3′; *SNAI2* forward 5′-TGTCATACCACAACCAGAGA-3′ and *SNAI2* reverse 5′- CTTGGAGGAGGTGTCAGAT-3′; *SOX2* forward 5′- AGAGAGAAAGAAAGGGAGAGA- 3′ and *SOX2* reverse 5′-AATCAGGCGAAGAATAATTTGG-3′; *TWIST1* forward 5′- CGGAGACCTAGATGTCATT-3′ and *TWIST1* reverse 5′-CTGTCTCGCTTTCTCTTTT-3′; *TWIST2* forward 5′- AACTGGACCAAGGCTCTC -3′ and *TWIST2* reverse 5′- GCGGCGTGAAAGTAAGAAT-3′; *ZEB1* forward 5′-CCAACAGACCAGACAGTG-3′ and *ZEB1* reverse 5′-TGACTCGCATTCATCATCTT-3′; *ZEB2* forward 5′- CGGAGACTTCAAGGTATAATCTATC-3′ and *ZEB2* reverse 5′- GTTACGCCTCTTCTAATGACAT-3′; *HPRT* forward 5′- AGACTTTGCTTTCCTTGGTCAGG-3′ and *HPRT* reverse 5′- GTCTGGCTTATATCCAACACTTCG-3′; *CD44v6* forward 5′- AGGAACAGTGGTTTGGCAAC-3′ and *CD44v6* reverse 5′- CGAATGGGAGTCTTCTCTGG-3′; *OCT4A* forward 5′-CCCCTGGTGCCGTGAA-3′ and *OCT4A* reverse 5′-GCAAATTGCTCGAGTTCTTTCTG-3′. Primers for *WSX-1 (IL27RA)* (product number QT00023373), *BMI1* (product number QT00052654), *c-MET* (product number QT00023408), *C-MYC* (product number QT00035406), *KLF4* (product number QT00061033), *NOTCH1* (product number QT01005109), and *SHH* (product number QT01156799), *SOX9* (product number QT00001498), were purchased from Qiagen.

The housekeeping gene hypoxanthine phosphoribosyltransferase 1 (*HPRT*) was used as endogenous control. Real-Time RT-PCR was performed using the MiniOpticon System (Bio-Rad) with SYBR green fluorophore under the following conditions: denaturation at 95°C for 3 minutes followed by 40 amplification cycles (denaturation at 95°C for 10 seconds and annealing/extension at 60°C for 30 seconds). Melting curve analysis was performed to assess the specificity of PCR products (65°C to 90°C at a ramp rate of 0.3°C every 5 seconds). The efficiency of reaction for each target was evaluated by amplifying serial dilutions of cDNA. Relative quantification of mRNA was performed according to the comparative threshold cycle method with HPRT as calibrator, using the software "Gene Expression Analysis for iCycler iQ® Real-Time PCR Detection System" (Bio-Rad). The samples were processed in triplicate and wells without added cDNA served as negative controls.

REFERENCES

S1. Landis JR, Koch GG. The measurement of observer agreement for categorical data. Biometrics. 1977; 33:159–174.

S2. Ghatak S, Bogatkevich GS, Atnelishvili I, Akter T, Feghali-Bostwick C, Hoffman S, Fresco VM, Fuchs JC, Visconti RP, Markwald RR, et al. Overexpression of c-Met and CD44v6 receptors contributes to autocrine TGF-β1 signaling in interstitial lung disease. J Biol Chem. 2014; 289:7856–72.

S3. Wang X, Zhao Y, Xiao Z, Chen B, Wei Z, Wang B, Zhang J, Han J, Gao Y, Li L, et al. Alternative translation of OCT4 by an internal ribosome entry site and its novel function in stress response. Stem Cells. 2009; 27:1265–75.

Supplementary Table S1: Antibodies used in immunostaining

Antibody	Clone	Origin	Source
On human tissue			
CD68	PG-M1	Mouse	Dako (Glostrup, DK)
CD15	Carb-3	Mouse	Dako
CD11c	EP1347Y	Rabbit	Abcam (Cambridge, UK)
WSX-1 (IL27Rα)		Rabbit	Novus Biologicals (Cambridge, UK)
On human xenograft			
CD31	SZ31	Rat	Dianova (Hamburg, Germany)
Laminin		Rabbit	BioGenex (Fremont, CA, USA)
IFNγ	LLO6Z	Mouse	Santa Cruz (Santa Cruz, CA, USA)
SNAI1		Rabbit	Santa Cruz
TRAIL		Goat	Santa Cruz
SOX9		Rabbit	Santa Cruz
SOX2	57CT23.3.4	Mouse	Abcam
KLF4	EPR3550(2)	Rabbit	Abcam
Nestin		Rabbit	Abcam
SHH	EP1190Y	Rabbit	Abcam
CXCL3		Rabbit	ProteinTech (Manchester, UK)
OCT4A	C52G3	Rabbit	Cell Signaling (Danvers, MA, USA)
NOTCH1	3E12	Mouse	Novus Biologicals
E-Cadherin	NCH-38	Mouse	Dako
SNAI2	1A6	Mouse	OriGene (Rockville, MD, USA)
ZEB1	7E12	Mouse	OriGene
TNFα*	MP6-XT22	Rat	BD Pharmingen (San Jose, CA,U.S.A)
CD11b/CD18 (Mac-1)*	M1/70.5	Rat	Harlan Sera Lab (Crawley Down, UK)
Granulocytes*	RB6-8C5	Rat	American Type Culture Collection

* Used on frozen sections.
